# FDX1 expression predicts favourable prognosis in clear cell renal cell carcinoma identified by bioinformatics and tissue microarray analysis

**DOI:** 10.3389/fgene.2022.994741

**Published:** 2022-09-16

**Authors:** Xing Huang, Tao Wang, Jiali Ye, Huayi Feng, Xiangyi Zhang, Xin Ma, Baojun Wang, Yan Huang, Xu Zhang

**Affiliations:** ^1^ Senior Department of Urology, The Third Medical Centre of PLA General Hospital, Beijing, China; ^2^ Medical School of Chinese PLA, Beijing, China

**Keywords:** ferredoxin 1, clear cell renal cell carcinoma, cuproptosis, metastasis, tumourigenesis, tissue microarray analyses, prognostic biomarker

## Abstract

Ferredoxin 1 (FDX1), an iron-sulphur protein, is responsible for electron transfer in a range of metabolic redox reactions. Clear cell renal cell carcinoma (ccRCC) is an aggressive cancer characterised by metabolic reprogramming, and FDX1 is a critical regulator of cuproptosis. However, the expression profile and prognostic value of FDX1 associated with clinicopathological features in ccRCC remain largely unelucidated. In this study, we integrated a series of public bioinformatic analysis to explore the mRNA and protein profiles of FDX1 across human cancers and cell lines and validated its expression and prognostic value, especially in ccRCC. In this study, FDX1 mRNA and protein expression were aberrantly downregulated and associated with ccRCC grade, stage, and nodal metastasis, whereas in adjacent non-tumour kidney tissue, it was abundantly expressed and cytoplasmically localised in renal tubular epithelial cells. Multivariate analysis indicated that low FDX1 expression contributed to unfavourable overall and disease-free survival. The functional enrichment of FDX1 co-expressed genes in ccRCC involved mainly mitochondrial dysfunction in various metabolic processes and biological oxidation, besides iron-sulphur cluster biogenesis. Furthermore, FDX1 modulates immunological infiltration to affect prognosis. Thus, FDX1 downregulation is mechanistically because of ccRCC tumourigenesis and is a promising prognostic biomarker to stratify patients with ccRCC.

## Introduction

In 2020, kidney cancer was the 16th most prevalent cancer type (431,288 new cases) worldwide, with high mortality (179,368 new deaths) (Sung et al., 2021). Clear cell renal cell carcinoma (ccRCC) is the most prevalent histological form of kidney cancer (approximately 75%) and is derived from the proximal nephric tubule (Hsieh et al., 2017). ccRCC is characterised by chromosome 3p loss of heterozygosity and von Hippel-Lindau mutations, which contribute to aberrant accumulation of many hypoxia-inducible factors. Regardless of oxygen availability, hypoxia-inducible factors promote gene transcription involved in neovasculogenesis, intratumoural heterogeneity, cell survival, and dysregulated cellular metabolism during glycolysis and lipolysis (Koppenol et al., 2011; Hsieh et al., 2017; Jonasch et al., 2021). Aberrant lipid and glycogen deposition also define ccRCC as a metabolic disease (Linehan et al., 2010; Du et al., 2017). Because of the genotypic and phenotypic characteristics of ccRCC, there remains a lack of appropriate biomarkers for reliable indication of prognostic or therapeutic results.

Deregulation of cellular metabolism is considered a hallmark of cancer (Hanahan, 2022). Emerging evidence has demonstrated that impaired mitochondrial metabolism results in tumourigenesis and the development of ccRCC, and inhibition of mitochondrial metabolism is exploited as an attractive therapeutic approach for cancer treatment (Porporato et al., 2018; Vasan et al., 2020; Stine et al., 2022). However, directly targeting mitochondrial respiratory activity could be a major challenge. The development of antimitochondrial metal complexes presents advantages such as bypassing cancer resistance, increasing selectivity, and re-activating cell-death programs (Erxleben, 2019; Stine et al., 2022). Copper-binding compounds can induce Cu-dependent cytotoxicity, emerging as a unique form of regulated cell death (RCD) (Tsvetkov et al., 2019). This novel RCD revealed that copper directly targets the lipoylated components of the tricarboxylic acid cycle to induce cuproptosis (Tsvetkov et al., 2022). Ferredoxin 1 (FDX1), an upstream regulator of protein lipoylation, is the direct target of copper ionophore elesclomol (Tsvetkov et al., 2019; Tsvetkov et al., 2022).

Human ferredoxin 1 (FDX1) and its paralogue form ferredoxin 2 (FDX2) are present in the mitochondrial matrix. Ferredoxin 1 functions in the biosynthesis of a small iron-sulphur (Fe-S) cluster that transfers electrons from NADPH through ferredoxin reductase (FDXR) to mitochondrial cytochrome P450 (CYP) for steroidogenesis (Sheftel et al., 2010; Strushkevich et al., 2011). Iron-sulphur [Fe-S] clusters are evolutionarily conserved in all living organisms and play fundamental roles in various biochemical processes, including the mitochondrial respiratory chain, central metabolism, redox catalysis, and regulation of gene expression (Fontecave, 2006; Py and Barras, 2010). The mechanism underlying the role of FDX1 in tumour development remains unclear. Limited findings on lung adenocarcinoma suggest that the function of FDX1 in tumourigenesis is related to the metabolism of glucose, amino acids, and fatty acid oxidation (Zhang et al., 2021). Another bioinformatic analysis study only referred to the cuproptosis-related gene signature for the prediction of the prognosis of ccRCC without experimental or human validation (Bian et al., 2022). Because ccRCC is a highly associated metabolic disease with mitochondrial function, the role of FDX1 in ccRCC is largely unknown.

In this study, transcriptome expression datasets and related clinical features were acquired from The Cancer Genome Atlas (TCGA) to investigate FDX1 altered expression and prognosis in pan-cancer and ccRCC. Tissue microarrays constructed from ccRCC samples by immunohistochemistry were used to determine the differential expression status of FDX1 associated with clinicopathological characteristics, and a Cox regression model was built for survival analysis. Furthermore, clinical human ccRCC, paired normal kidney samples, and ccRCC cell lines were used to validate the expression of FDX1 protein. These results provide a fundamental FDX1 expression profile to predict copper chelator sensitivity and suggest that FDX1 is a promising novel biomarker with an effective prognostic value for ccRCC.

## Materials and methods

### Patients and tumour specimens

From August 2012 to December 2019, paraffin-embedded specimens of paired tumours and adjacent normal tissues from 199 patients with detailed follow-up information were collected for tissue microarray construction. The other 27 pairs of tumours and para-tumorous tissue of ccRCC were immediately snap-frozen in liquid nitrogen and then extracted for qRT-PCR analysis. A total of 226 enrolled patients underwent partial or total ccRCC resection at the Chinese People’s Liberation Army General Hospital. Written informed consent was obtained from all participants. The study was conducted in accordance with the protocol approved by the Protection of Human Subjects Committee of the Chinese People’s Liberation Army General Hospital (No. S2021-176-01). The clinicopathological characteristics of patients with ccRCC are summarised in Table 1. The TNM classification was confirmed by a pathologist at our institution and staged according to the manual of the American Joint Committee on Cancer (eighth edition). The inclusion criteria were as follows: conventional surgical treatment without radiation or chemotherapy, histopathologically diagnosed clear cell renal cell carcinoma, postoperative follow-up for at least 12 months, and comprehensive clinical medical records. Patients with severe underlying conditions, inadequate data, benign renal tumours, Xp11.2 translocation/TFE3 fusion gene-related renal carcinoma, papillary carcinoma, and other non-clear cell carcinomas were excluded.

**TABLE 1:** Correlation between FDX1 expression and clinical characteristics in 199 patients with ccRCC.

Characteristics	Numbers	FDX1		χ^2^ value	*p* Value
		Low	High		
Numbers	199	34	165		
Age(years)					
≤60	134	20	114	1.351	0.315
>60	65	14	51		
Sex					
Male	152	29	123	1.805	0.267
Female	47	5	42		
BMI					
<28	154	29	125	1.465	0.267
≥28	45	5	40		
T stage					
T1	153	20	133	7.526	**0.012**
T2-T4	46	14	32		
N stage					
N0	192	31	161	1.777	0.098
N1	7	3	4		
M stage					
M0	170	24	146	5.887	**0.014**
M1	29	10	19		
AJCC stage					
I-II	153	18	135	13.227	**0.001**
III-IV	46	16	30		
ISUP Grade					
I-II/Low	148	17	131	12.779	**0.001**
III-IV/High	51	17	34		

*p* value <0.05 marked in bold font shows statistical significance. BMI, Body Mass Index; T, Primary tumour size; N, Lymph node metastasis; M, Distant metastasis.

### Cell lines

Cell lines 293T, ACHN, Caki-1, A498, overall survival (OS)-RC-2, 769-P, and 786-O were purchased from the National Platform for Experimental Cell Resources for Sci-Tech (Beijing, China). The SN12-PM6 cell line was provided by Dr. X.P. Zhang (Tongji Medical College, Huazhong University of Science and Technology, China). All cell lines were cultured in Dulbecco’s Modified Eagle’s Medium, Minimum Essential Media, Roswell Park Memorial Institute 1640 medium or McCoy’s 5A (Procell, China) supplemented with 10% foetal bovine serum (FBS) (164210-50, Procell, China) and 1% penicillin G sodium/streptomycin (PB180120, Procell, China). Cell lines were incubated under a humidified 37°C incubator containing 5% CO_2_.

### Western blot analysis

Total protein from tissues and cells was isolated using RIPA lysis buffer containing a protease inhibitor cocktail (#5871, Cell Signalling Technology, Inc.). The BCA method was used for protein quantification. The quantified protein (80 µg) was separated by 12% SDS/PAGE gel electrophoresis and then transferred to 0.22 μm polyvinylidene fluoride membranes (Direct-Q, Millipore Sigma). After blocking the membranes with 5% non-fat milk for 1.5 h at 37°C, they were incubated with the respective primary antibodies for 16 h at 4°C. Primary antibodies were FDX1 Polyclonal antibody (1:1,000, 12592-1-AP, Proteintech) and β-tubulin (1:3,000, BE0025, Bioeasytech, Inc.). Secondary antibodies were goat anti-rabbit IgG HRP-linked antibody (1:5,000, BE0101, Bioeasytech, Inc.) and goat anti-mouse IgG HRP-linked antibody (1:5,000, BE0102, Bioeasytech, Inc.). Immunoblotting was visualised and quantified using the Tanon Gel Image System (Tanon-5200, Biotanon, Shanghai, China).

### Reverse transcription-quantitative polymerase chain reaction (RT-qPCR)

Cells and tissues were lysed using the TRIzol^®^ reagent (Invitrogen; Thermo Fisher Scientific, Inc.). Total RNA was extracted and reverse-transcribed using the iScript cDNA synthesis kit (170-8891, Bio-Rad Inc.) at 42°C for 15 min. Real-time RT-qPCR was performed using iTaq universal SYBR Green supermix (1725122, Bio-Rad Inc.). Subsequent quantitative polymerase chain reaction (qPCR) analyses for each cDNA gene were quantified under predefined conditions as follows: polymerase activation and pre-denaturation at 95°C for 25 s; 40 amplification cycles of denaturation (94°C for 15 s), annealing/extension (60°C for 60 s), and the melt curve analysis step. Reactions were conducted using ABI QuantStudio 5 (Applied Biosystems; Thermo Fisher Scientific, Inc.) (Applied Biosystems; Thermo Fisher Scientific, Inc.). Homo sapiens peptidylprolyl isomerase A (PPIA) was used as an internal reference. The validated primers were FDX1 sense, 5′- CTG​GCT​TGT​TCA​ACC​TGT​CAC​C-3′ and anti-sense, 5′- GAT​TTG​GCA​GCC​CAA​CCG​TGA​T-3′; PPIA sense, 5′-GTG​TTC​TTC​GAC​ATT​GCC​GTC-3′ and anti-sense, 5′-TGC​ACG​ATC​AGG​GGT​AAA​CA-3′. Data were calculated using the 2-ΔΔCq method (Livak and Schmittgen, 2001).

### Tissue microarray (TMA) and immunohistochemistry (IHC)

TMAs containing 199 paired formalin-fixed paraffin-embedded ccRCC and adjacent normal tissue samples were constructed. IHC for FDX1 expression in TMAs was performed using a standard protocol (19). The tissue microarray was constructed manually, and a 2 mm cylindrical core sample from tissue donor blocks (form cohort, *see* Table 1) was placed in a prepared 6 × 10 array of recipient wax blocks with 1.5 mm hole spacing. The recipient wax block was arranged with a maximum of 30 matched pairs of tumours and adjacent tumour tissues. Paraffin-embedded 5 µm thick tissue sections were de-paraffinised by the following steps: 2 × 10 min in xylol; 2 × 5 min in 100% ethanol; and 90, 80, and 70% ethanol for 5 min each. The sections were then placed in 10 mM citric acid buffer for antigen retrieval and boiled in a microwave oven at 100°C for 15 min. The slides were incubated with rabbit anti-FDX1 antibody overnight at a dilution of 1:200 at 4°C, followed by incubation with a secondary anti-rabbit antibody (PV-9001, OriGene Technologies, Inc.) at RT. Each TMA spot and IHC slide were photographed using the TissueFAXS imaging system (TissueGnostics GmbH, Austria) and evaluated by staining intensity: 0 (no cytoplasmic staining), 1 (faint positive), 2 (moderate positive), or 3 (intense positive). In each TMA section, the percentage of tumour cells with positive cytoplasmic staining was scored as 0 (<10%), 1 (10–25%), 2 (25–50%), 3 (50–75%), and 4 (>75%). The total IHC score was then calculated as follows: intensity of staining × percentage of staining.

### RNA-seq and proteomic analysis of FDX1 expression

We investigated the molecular characterisation of gene expression, tumour-immune interactions, and clinical outcomes using the tumour-immune estimation resource (TIMER) (Li et al., 2017). The Gene-DE (differential gene expression) module in TIMER2.0 (http://timer.comp-genomics.org/) was used to test differential expression of FDX1 mRNA between tumours and neighbouring normal tissues in all cancers. The Wilcoxon test was used for statistical calculations. Spearman’s correlation test was used to examine the relationships between FDX1 differential expression and clinical characteristics, such as OS, cancer stage, and tumour grade, in all TCGA tumours in the TISIDB (http://cis.hku.hk/TISIDB/index.php) (Ru et al., 2019).

The UALCAN (http://ualcan.path.uab.edu/index.html) interface was used to obtain data on FDX1 expression from the ccRCC subgroup in pathological stages, grades, and nodal metastases (Chandrashekar et al., 2017). Besides RNA-seq data, the protein expression level of FDX1 between primary tumour tissues and normal tissues was analysed using cancer omics data from the updated UALCAN (Chandrashekar et al., 2022). The Clinical Proteomic Tumour Analysis Consortium (CPTAC) provided proteomic data from mass spectrometry. Z-values, which represent standard deviations from the median across samples for the 10 cancer types, were used to analyse the differences in protein expression.

Quantitative profiling of RNA-seq in 1000 human cancer cell lines and proteomic information in 375 cancer cell lines were acquired from the Cancer Cell Line Encyclopedia (CCLE, https://sites.broadinstitute.org/ccle/) (Nusinow et al., 2020). The DepMap (Cancer Dependency Map) portal was then used to obtain FDX1 differential expression across cell lines using the dataset “DepMap Public 22Q2,” which contains data from the project Achilles and the CCLE. Applying the R packages “ggplot2” and “ggpubr,” FDX1 mRNA and protein expression differences across cancer cell lines were visualised in box plots, and FDX1 mRNA expression profiles in Renal cancer cells (RCC) are shown in the dot plot.

### Survival analysis of TCGA cancers

To examine the prognostic value of OS and disease-free survival (DFS) associated with FDX1 differential expression in diverse malignancies, we performed survival analysis using GEPIA2.0 (Gene Expression Profiling Interactive Analysis, http://gepia2.cancer-pku.cn/#index). GEPIA2, an interactive resource for gene expression analysis, was based on 9736 cancer and 8587 normal samples from the TCGA and GTEx databases (Li et al., 2021). We categorised samples according to differential expression of FDX1, and the group cutoff was defined as the median with a cutoff of 50% high and a cutoff of 50% low for all 33 types of TCGA cancer. The survival map of FDX1 is shown according to the colour reflected by the log10 (HR) value. Different Kaplan–Meier curves with significance for survival were plotted for different types of cancer. Furthermore, in our cohort of 199 patients with ccRCC, patients were classified into high and low FDX1 expression groups according to the protein expression level semi-quantified by the IHC score. The Kaplan–Meier survival curve for each group was determined using the log-rank test.

### Functional enrichment analysis

Differentially expressed genes (DEGs) related to FDX1 in ccRCC were gathered from the LinkedOmics database (http://www.linkedomics.org/login.php), which provided a unique platform to access, analyse, and compare cancer multi-omics data from all types of cancer TCGA and 10 CPTAC cancer cohorts (Vasaikar et al., 2018). Significantly upregulated and downregulated DEGs related to FDX1 were aggregated for gene set enrichment analysis, using the Link-interpreter module. Gene Ontology (GO) annotations on biological processes (BP), cellular components (CC), molecular function (MF), and Kyoto Encyclopedia of Genes and Genomes (KEGG) analysis were assessed respectively, to discover enrichment functions related to FDX1 co-expressed gene sets. The Reactome pathway database (https://reactome.org/) is an effective platform for visualising human pathways and functional enrichment analysis of the top 100 FDX1 positively correlated genes (Fabregat et al., 2017).

### Protein-protein interaction (PPI) networks

We used the Search Tool for the Retrieval of Interacting Genes/Proteins database (STRING, https://cn.string-db.org/) to query the FDX1 protein in Homo sapiens. Using functional association data, we used GeneMANIA (http://genemania.org/) to predict the function of FDX1 and its interacting genes based on GO annotations to construct the PPI network (Warde-Farley et al., 2010). Jvenn (http://jvenn.toulouse.inra.fr/app/example.html) was used to draw Venn diagrams to determine functional proteins of common interaction.

### Immune cell infiltration analysis

TISIDB (http://cis.hku.hk/TISIDB/index.php) is a comprehensive online resource that evaluates interactions between tumour and immune system (Ru et al., 2019). This database was used to examine the associations between FDX1 expression and the abundance of TILs in pan-cancers and ccRCC. The correlation between FDX1 expression and TILs was determined using Spearman’s correlation test.

### Statistical analysis

A Wilcoxon matched-pair signed-rank test was performed to assess differences in relative mRNA levels between patient groups. The chi-square test was used to investigate the relationship between FDX1 expression levels and clinicopathological variables. Student’s t test was used for comparison between the two groups. Measurement data between three or more groups was examined by one-way ANOVA, followed by Tukey’s *post hoc* test. The Cox proportional hazards regression model was used to determine risk factors. Statistical calculations were performed using IBM SPSS Statistics 26.

## Results

### Downregulated FDX1 expression is significantly associated with clinical characteristics in ccRCC

**FIGURE 1:**
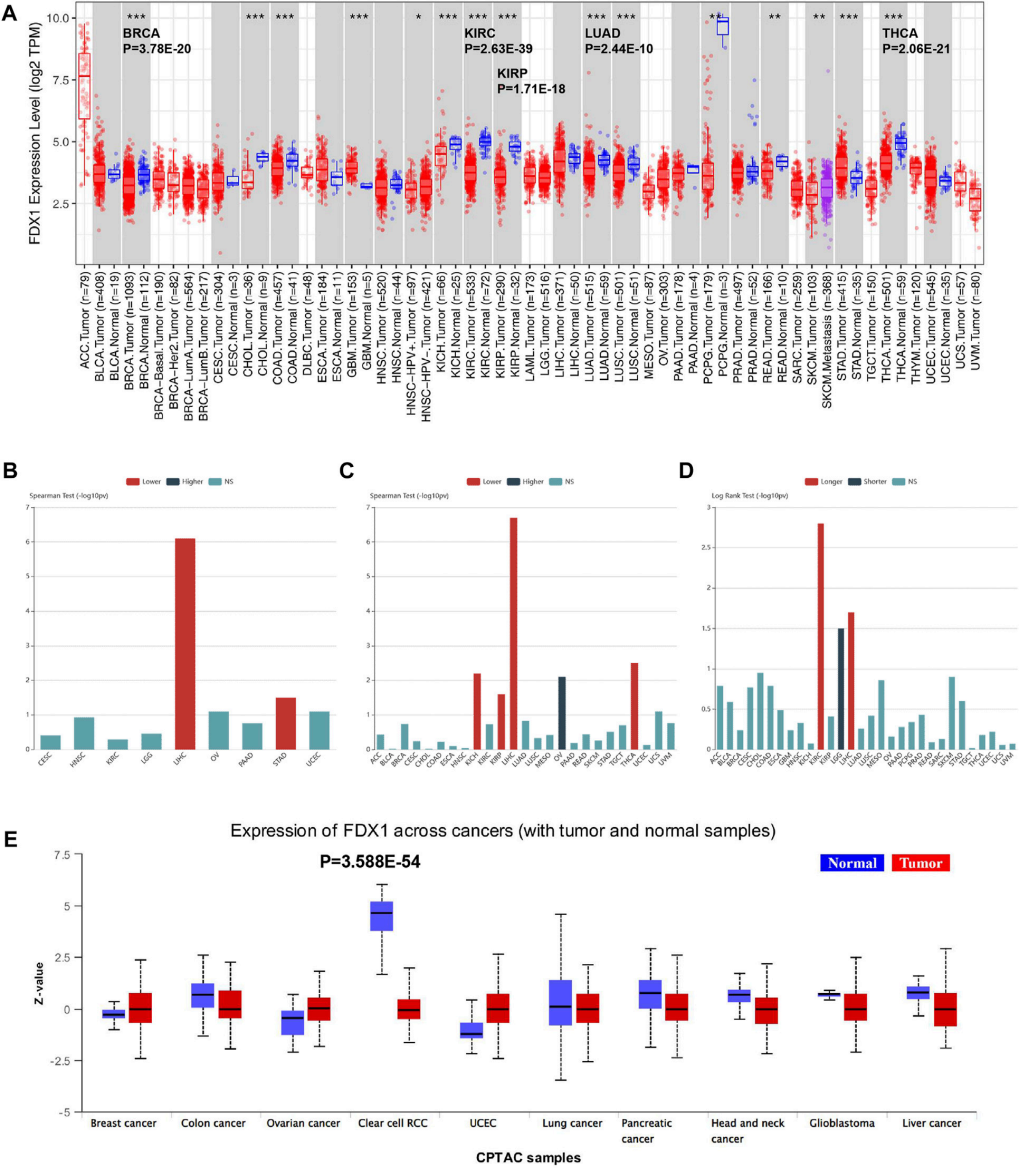
TCGA study of FDX1 RNA and protein expression and correlation with clinical characteristics. **(A)** Pan-cancer relative of human FDX1 mRNA expression across 33 TCGA cancers in TIMER. The statistical significance is annotated by the number of stars (*p < 0.05, **p < 0.01, ***p < 0.001 and ****p < 0.0001). **(B)** Associations between FDX1 mRNA level and patient pathological grade in different cancers in TISIDB. **(C)** Significant associations between FDX1 expression and stage across cancers (Spearman correlation test *p* 0.05). **(D)** Associations between FDX1 expression level and overall survival among human cancers. **(E)** FDX protein expression of tumour vs. normal samples across human tumours based on CPTAC (clinical proteomic tumour analysis consortium) analysis. Z-values show standard deviations from the median across samples for the specified cancer type. NS, not significant.

To determine the expression profile of FDX1 in all 33 TCGA cancer types, we performed TCGA analysis of FDX1 mRNA expression levels measured by log2 TPM (Transcripts Per Kilobase Million) in the RNA-seq database (Figure 1A). The bar chart in Figure 1A reveals that FDX1 mRNA expression in normal and primary tumour tissues was downregulated usually. The differential expression of FDX1 was significant in ccRCC compared with that in other cancer types (*p =* 2.63E–39). The other main expression differences of FDX1 were in THCA (*p =* 2.06E–21), BRCA (*p =* 3.78E–20), KIRP (*p =* 1.71E–18), and LUAD (*p =* 2.44E–10), with downregulated expression in human tumour tissue. Next, we used the Tumour-Immune System Interaction Database (TISIDB) to investigate the FDX1 association among clinical features across tumours. mRNA expression analysis based on (–log10PV) demonstrated that FDX1 mRNA levels were strongly associated with a lower pathological stage in LIHC and STAD (Figure 1B). Regarding tumour grade, FDX1 was associated with a lower grade in KICH, KIRP, LIHC, and THCA, whereas a higher grade was associated with OV (Figure 1C). Furthermore, survival outcome *via* the log-rank test illustrated FDX1 upregulated expression was predominantly associated with KIRC, indicating the longest survival significance among cancers (Figure 1D). Furthermore, FDX1 is a favourable biomarker for LIHC. However, unlike KIRC and LIHC, which showed a positive correlation, for FDX1, a hazardous signature was observed for LGG (Figure 1D).

To evaluate FDX1 protein expression levels and validate the consistency with mRNA expression profiles among cancer types, the CPTAC datasets with cancer proteomic data were used to assess protein expression. We compared normal and tumour tissues in 10 tumours (Figure 1E), and FDX1 protein level was defined as the most significant difference in KIRC (*p =* 3.588E–54). This finding implies that FDX1 might be involved in ccRCC tumourigenesis.

To investigate the difference in FDX1 expression in cancer cell lines, we obtained the mRNA and proteomic data of various cancer cell lines from DepMap. Across cancer cell lines, neuroblastoma displayed the lowest FDX1 mRNA expression level in the box plot; adrenal cancer had the highest expression (Supplementary Figure S1A). RCC exhibited a relatively low expression below the dashed lines, which indicated the median expression value of FDX1 mRNA of all cancer cell lines (Supplementary Figure S1A). Based on the proteomic assessment of FDX1 expression profiles in cancer cell lines, neuroblastoma, thyroid cancer, and kidney cancer were ranked as the top three cancers with the lowest expression (Supplementary Figure S1B). FDX1 mRNA expression was plotted in distinctive RCC cell lines, and RCC10RGB was identified as the cell line with the highest decrease (Supplementary Figure S1C).

Therefore, the expression of FDX1 at mRNA and protein levels was significantly correlated in RCC cell lines and human RCC, and the downregulated expression of FDX1 indicated the specificity of clinical and diagnostic values in ccRCC.

### Prognosis values and FDX1 risk ratio in pan-cancer

**FIGURE 2:**
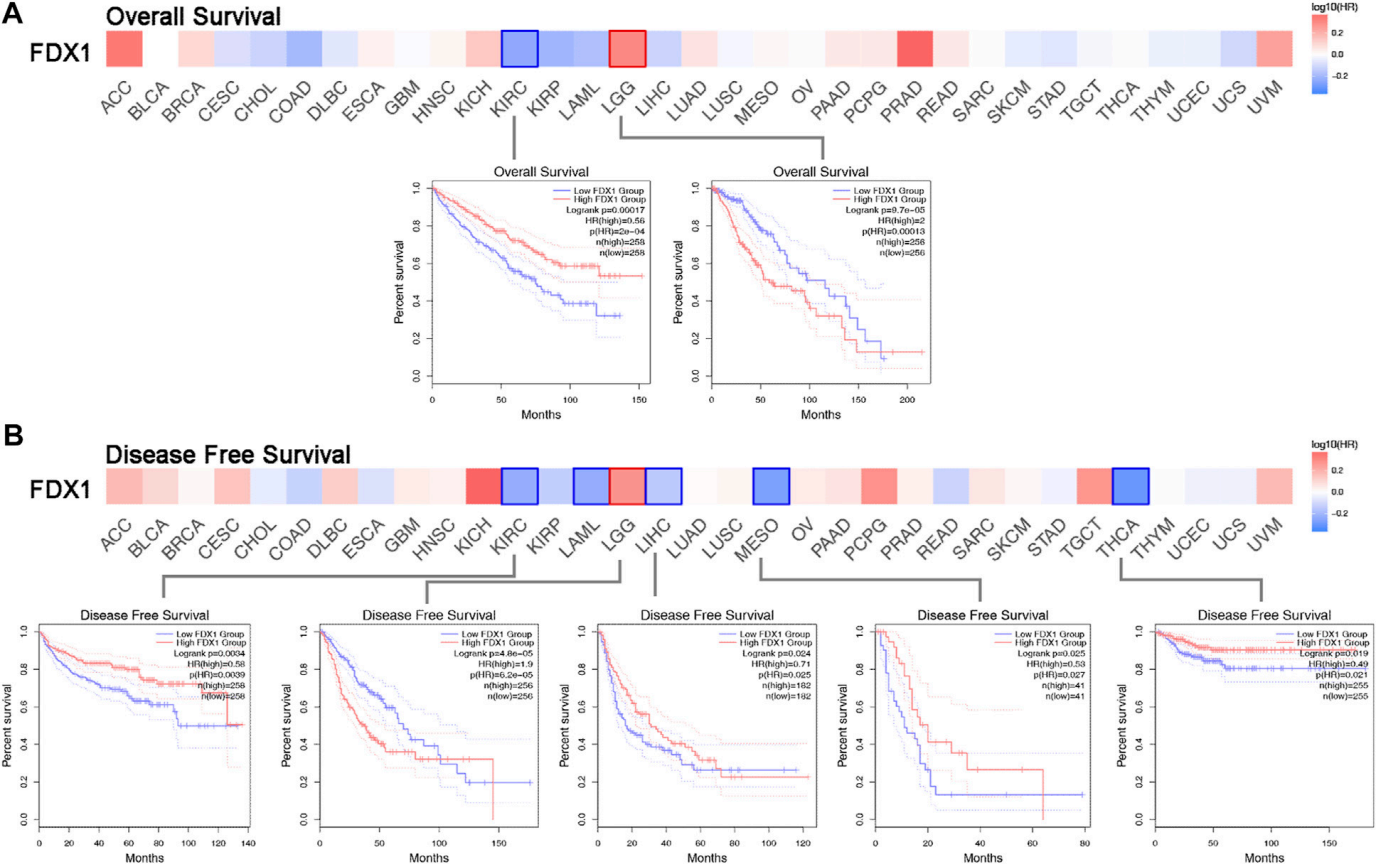
Survival hazard ratio between high and low FDX1 expression in multiple TCGA cancer types. **(A)** Significance map of the overall survival analysis based on FDX1 expression. The Kaplan-Meier survival curve depicts the significant survival contribution of FDX1 in KIRC and LGG. **(B)** Disease-free survival analysis on FDX1 expression significance map FDX1 expression in KIRC, LAML, LGG, LIHC, MESO, and THCA reveals significant survival contributions.

To acquire comprehensive prognostic values of FDX1 in pan-cancer, we used the TCGA and GEO datasets to present the correlation between FDX1 expression and prognosis. We then depicted the survival map of both OS and DFS using hazard ratios. The Kaplan–Meier survival curve demonstrated survival significance between the high and low FDX1 expression groups. The survival significance map of overall survival revealed that KIRC had a favourable OS in the high FDX1 expression group (HR = 0.56, *p =* 0.00017) (Figure 2A, left Kaplan–Meier curve), whereas LGG was associated with adverse OS in LGG (HR = 2, *p =* 9.7E–05) (Figure 2A, right Kaplan–Meier curve). Regarding DFS, there was an association between high FDX1 expression and satisfactory prognosis in TCGA cancers, such as KIRC (HR = 0.58, *p* = 4.8E–05), LIHC (HR = 0.71, *p* = 0.024), MESO (HR = 0.53, *p* = 0.025), and THCA (HR = 0.49, *p* = 0.019), and high FDX1 expression was regarded as a hazardous factor for LGG (HR = 1.9, *p* = 4.8E-05) (Figure 2B). The Kaplan–Meier survival curve for DFS was omitted for patients with AML without disease-free status. The FDX1 expression has a significant prognostic value for OS and DFS in patients with ccRCC.

### Downregulated FDX1 transcriptional expression and clinical characteristics in ccRCC

**FIGURE 3:**
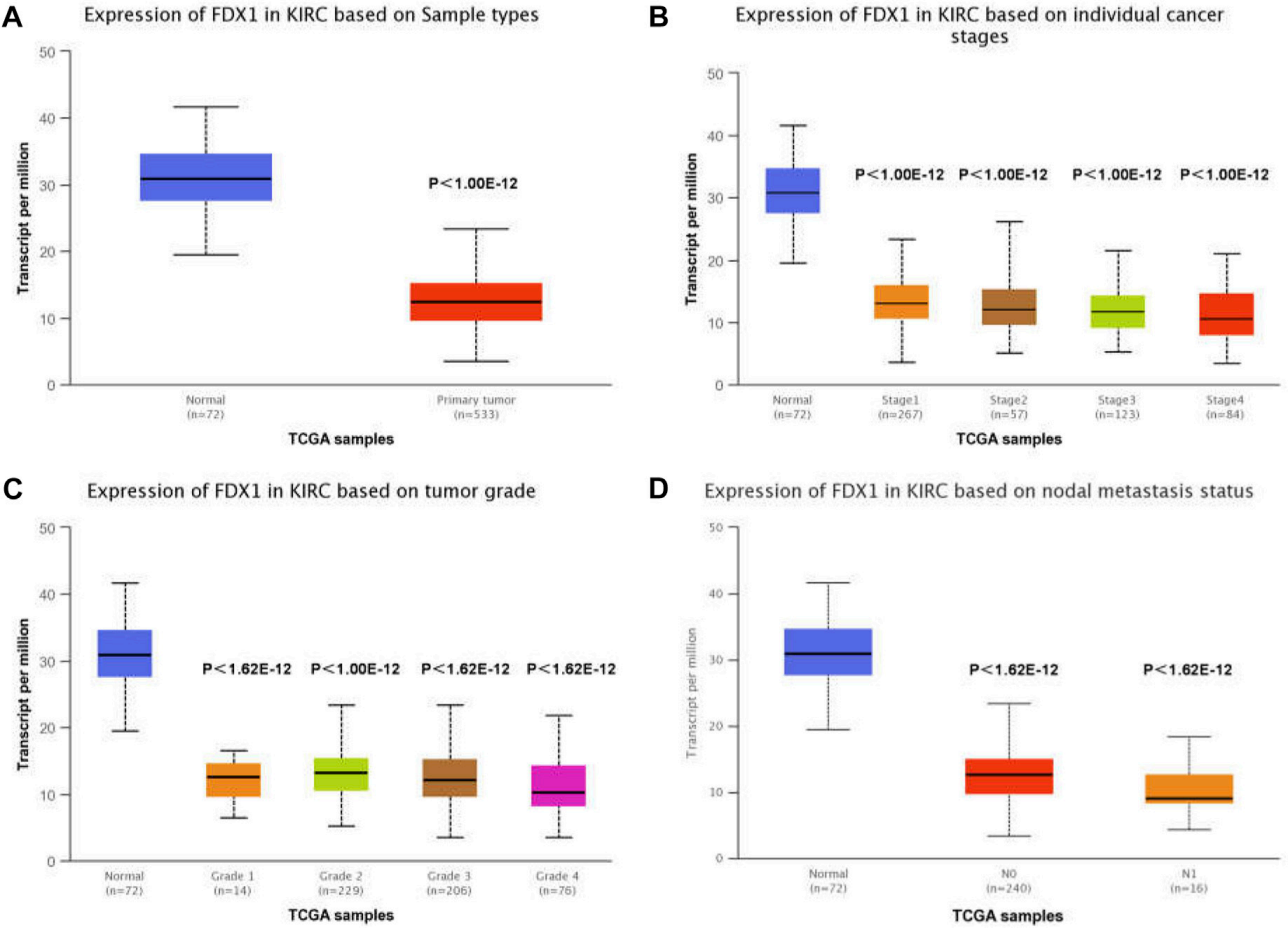
TCGA analysis of FDX1 demonstrates significantly reduced expression in ccRCC. **(A)** FDX1 expression in KIRC tissue compared to adjacent normal tissue. **(B)** Expression of FDX1 in different stages of ccRCC. **(C)** FDX1 expression in ccRCC of various tumour grades. **(D)** Expression of FDX1 in ccRCC depends on the presence or absence of nodal metastases.

To determine the association between FDX1 expression and clinicopathological characteristics in ccRCC, we used the UALCAN interactive web platform to analyse TCGA RNA-seq data [3]. The relative expression of FDX1 was high in normal kidney samples, but significantly downregulated in primary ccRCC (Figure 3A). In this study, FDX1 was significantly downregulated at all ccRCC stages (Figure 3B). A comparison of mRNA expression among groups of tumour stages demonstrated that the ccRCCs of stages 3 and 4 showed the lowest FDX1 expression (Figure 3B). In addition, regarding pathological grade, FDX1 expression was inhibited in tumour groups ranging from grades 1 to 4, and grade 4 was associated with the lowest FDX1 expression (*p <* 0.01, grade 3 vs. grade 4) (Figure 3C). Furthermore, to evaluate whether the status of nodal metastases could affect FDX1 expression, we compared groups of patients with or without nodal metastases. The box-whisker plots (Figure 3D) demonstrated a further decrease in FDX1 expression in patients with nodal metastasis (*p <* 0.05, N0 vs. N1 group). Collectively, the aggressive clinicopathological characteristics of ccRCC could further inhibit FDX1 expression, and downregulated expression in primary ccRCC has essential implications for the underlying function of FDX1 in tumorigenesis.

### Reduced FDX1 expression in tumour cell lines and human ccRCC analysed by tissue microarray

**FIGURE 4:**
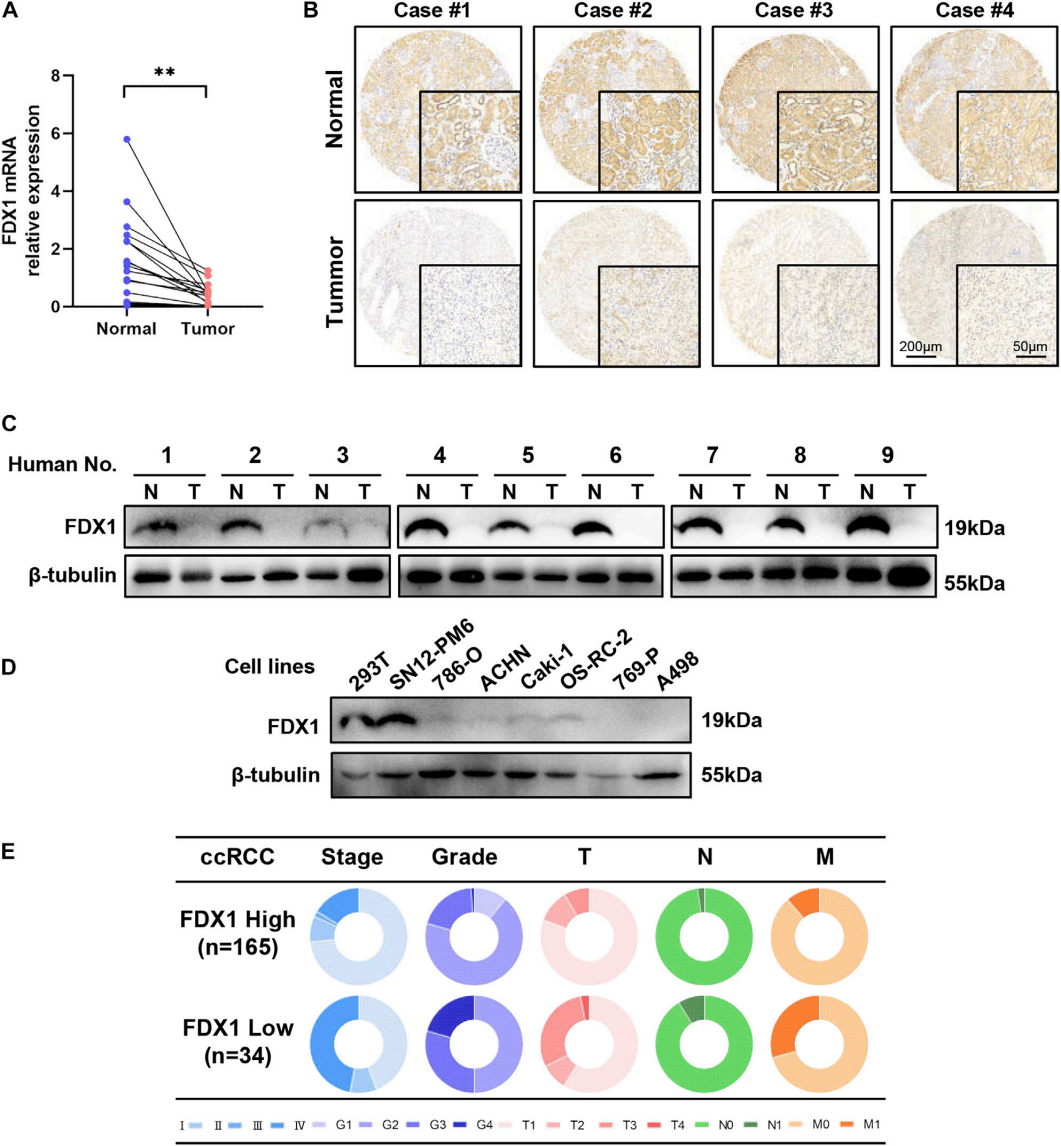
FDX1 is overexpressed in normal tissues and downregulated in ccRCC and cell lines. **(A)** RT-qPCR analysis of FDX1 mRNA expression between normal and tumour tissues in 27 pairs of patients’ tissues. **(B)** Tissue microarray analysis by IHC was performed to evaluate the protein expression level of FDX1 in para-tumorous normal tissue and primary tumour tissue from 199 patients. **(C)** FDX1 protein expression between normal and cancerous specimens was investigated by a western blot analysis. **(D)** Immunoblotting assay of FDX1 protein expression in ccRCC cell lines and 239T control cell lines. **(E)** The circular pie chart illustrates the proportional fluctuation of clinical parameters (including AJCC stage, ISUP grade, tumour size, node, and metastasis stages) in the FDX1-high and FDX1-low expression groups in TMA with 199 patients with ccRCC.

To investigate the relative expression of FDX1, we performed RT-qPCR, western blotting, and immunohistochemistry (IHC) on TMA to quantify mRNA, protein, and histopathological protein expression levels in human tissues and cell lines. First, acquired cDNA isolated from 27 clinically resected ccRCC samples with paired tumours and adjacent normal tissues was used for RT-qPCR. FDX1 was considerably downregulated (*p <* 0.01) in ccRCC, which exhibited a clear and consistent decrease of mRNA expression levels in human ccRCC tissue (Figure 4A). IHC was performed to determine the expression levels of the FDX1 protein in samples from four patients with pairs of paratumoural tissues and primary tumour tissues (Figure 4B). IHC analysis demonstrated that FDX1 was abundantly expressed in normal kidney tissue with cytoplasmic expression, in the proximal and distal renal tubules, and less abundantly expressed in the collecting duct (Figure 4B). In tumorous tissue, FDX1 expression was considerably downregulated in all ccRCC specimens and exhibited the lowest staining intensity in Case 1, with stage 4 ccRCC (Figure 4B).

Next, we validated FDX1 expression using proteins from nine pairs of tissues. Western blotting indicated that FDX1 decreased in all nine ccRCC samples compared to adjacent normal samples (Figure 4C). The expression of the FDX1 protein was also consistent with transcriptional expression. Western blot analyses were performed on human ccRCC cell lines, and 293 T cells were used as controls. These findings revealed reduced expression of FDX1 in most ccRCC cell lines, except the SN12-PM6 cell line (Figure 4D), which demonstrated increased expression.

To evaluate the correlation between FDX1 expression and clinicopathological features, we performed a tissue microarray analysis by IHC using paraffin-embedded paired tissues from 199 patients with ccRCC at the Chinese People’s Liberation Army General Hospital. The patients were separated into high and low FDX1 groups according to the IHC score. IHC scores of 4 or less were assigned to the low FDX1 expression group and vice versa. For the expression groups of high FDX1 and low FDX1, the circular pie chart highlights the detailed proportion of each clinical feature (Figure 4E). Therefore, patients with relatively high FDX1 expression accounted for an increased early clinical proportion in the AJCC stage (blue colour), ISUP grade (purple colour), T (pink colour), N (green colour), and M (orange colour) stages, whereas patients with relatively low FDX1 expression were in more aggressive stages than the high FDX1 expression group (Figure 4E). The association between FDX1 expression and clinical features in patients with ccRCC is presented in Table 1. There was a significant relationship between low expression of FDX1 and clinical characteristics in stage T (*p =* 0.012), M (*p =* 0.014), AJCC (*p =* 0.001), and WHO/ISUP grade (*p =* 0.001). These results confirm that FDX1 expression decreases in most ccRCC cell lines, and FDX1 mRNA and protein expression were strongly downregulated in primary human ccRCC tissues associated with the aggressive stage of AJCC and ISUP grade.

### FDX1 is a favourable prognostic biomarker in ccRCC

**FIGURE 5:**
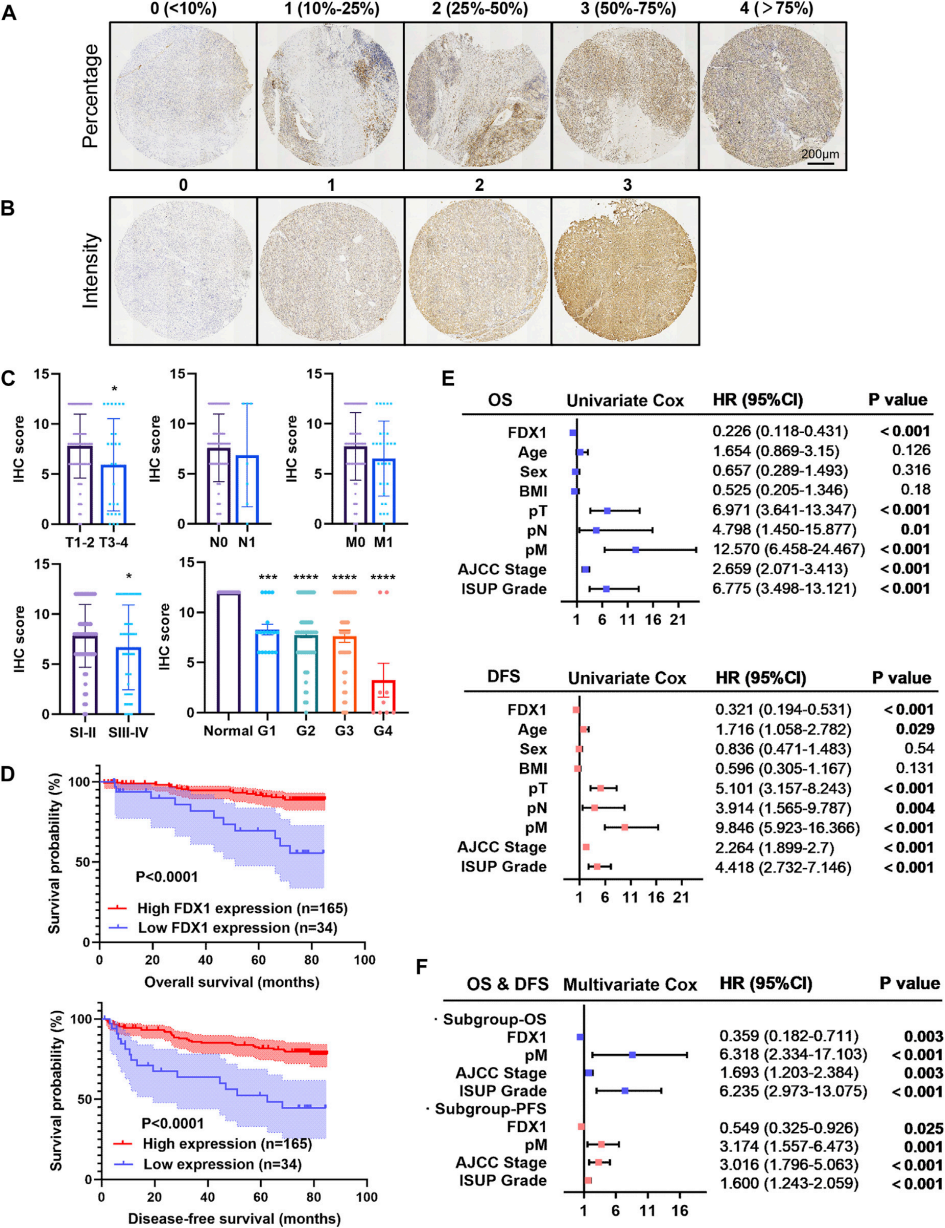
Tissue microarray analysis of FDX1 expression in 199 patients and its prognostic significance in ccRCC. **(A**,**B)** Immuno-histochemistry of TMA and scoring according to the positive stain percentage (score 0–4) and intensity (score 0–3). **(C)** Bar chart exhibited the quantification of the immunohistochemistry score between groups with a *p* value. **(D)** Kaplan-Meier survival analysis demonstrated the positive OS and DFS probability of ccRCC patients with a greater FDX1 expression. **(E)** Univariate Cox analysis for overall survival and disease-free survival in the cohort in relation to clinicopathological factors (including FDX1 expression, age, gender, BMI, pT, pN, pM, AJCC stage, and ISUP grade). **(F)** Forest plot for the multivariate Cox analysis for overall survival and disease-free survival. *p < 0.05, ***p < 0.001 and ****p < 0.0001 vs. para-tumour tissue.

To determine the association between FDX1 expression and clinicopathological characteristics and prognosis, we multiplied the FDX1 staining percentage (Figure 5A) by the staining intensity (Figure 5B) to obtain an IHC score for each TMA slide. Slides were associated with lower IHC scores in the T3-4 group, AJCC stage 3–4 group, and ISUP grade 1–4 groups (*p <* 0.05, Figure 5C), consistent with TCGA data mining of FDX1 mRNA expression based on tumour stage and grade (Figures 3B,C).

Furthermore, with a median follow-up time of 76.670 ± 0.483 months, Kaplan–Meier survival by log-rank analysis indicated a significantly favourable 5-year OS and DFS in the high expressed FDX1 group (*p <* 0.0001; Figure 5D). In forest plots with hazard ratios and 95% confidence intervals, a univariate Cox regression analysis was performed based on clinicopathological characteristics (Figure 5E). We identified FDX1 as a significant protective factor for both OS (HR, 0.226; *p <* 0.001) and DFS (HR, 0.321). Other clinical variables such as larger tumour size, nodal involvement, distal metastasis, higher AJCC stage, and elevated ISUP grade were hazardous factors for OS and DFS of patients with ccRCC (Figure 5E). Furthermore, we observed that high expression of FDX1 remained an independent protective factor for OS (*p =* 0.03; HR, 0.359) and DFS (*p =* 0.025; HR, 0.549) by stepwise multivariate Cox regression analysis (Figure 5F). Distal tumour metastasis, the AJCC stage and ISUP grade were identified as common risk factors for OS and DFS (Figure 5F). Therefore, the IHC analysis of FDX1 expression revealed that FDX1 functions in tumour progression and malignancy.

### Functional enrichment analysis of FDX1 and co-expressed genes

**FIGURE 6:**
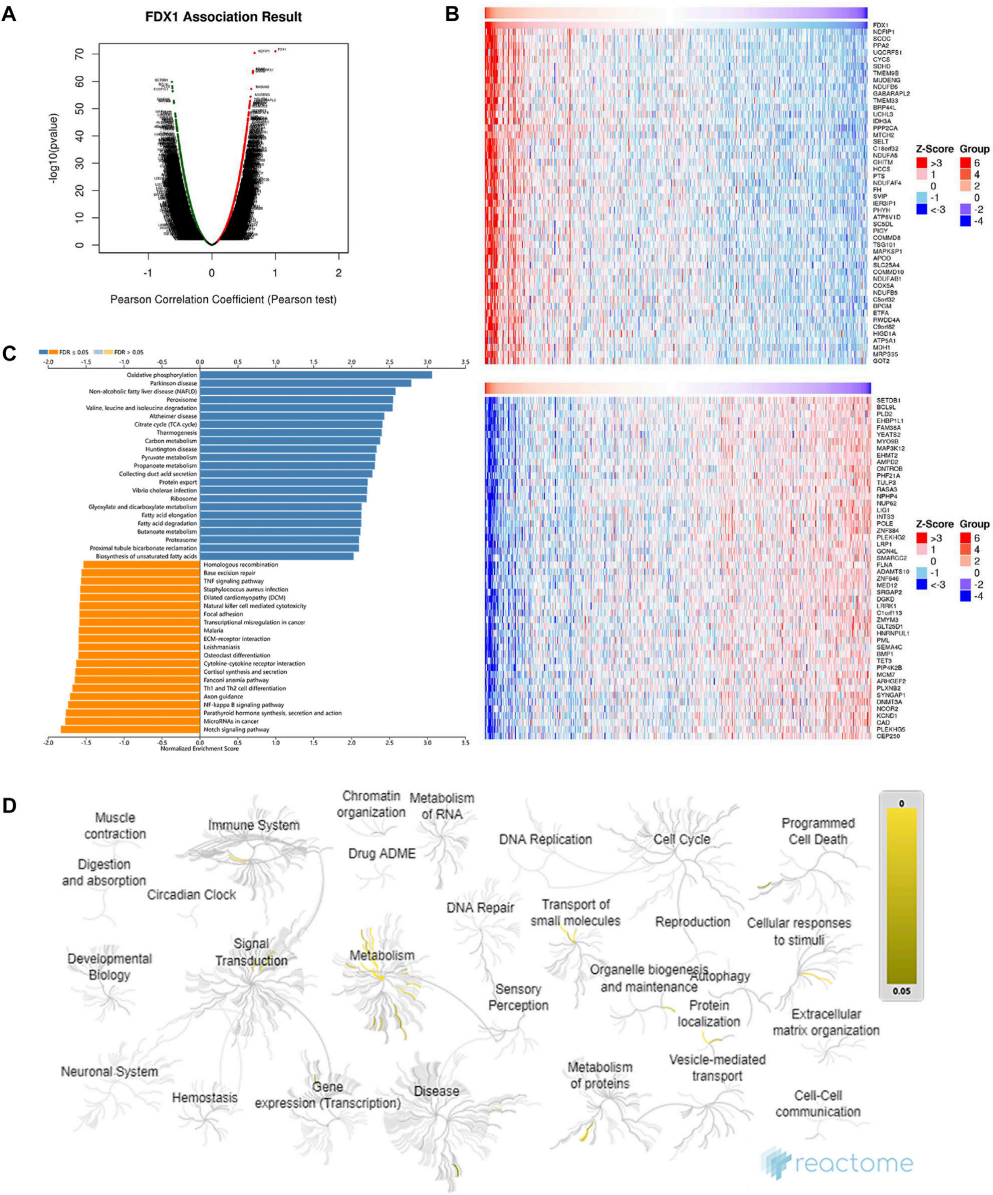
Functional enrichment analysis and co-expression profile highly associated with FDX1 expression in ccRCC. **(A)** Volcano plot of differentially expressed genes associated with FDX1 based on Pearson’s correlation coefficient analysis. Genes with a green dot were downregulated, whereas genes with a red dot were upregulated. **(B)** The top 50 co-expressed genes’ heatmaps show a positive (upper heatmap) and negative (lower heatmap) correlation with FDX1. **(C)** A bar diagram of the upregulated (blue bar) and downregulated (orange bar) signalling pathways revealed by KEGG analysis. **(D)** Functional enrichment of the top 100 genes positively correlated with FDX1 expression in the Reactome enrichment pathways with yellow gradient representing statistical significance (*p*-value).

To understand the function of FDX1 in the biological modulation of ccRCC, DEGs associated with FDX1 were analysed on the LinkOmics platform. We observed that 7439 genes were upregulated (red dot, Figure 6A) and 12,720 genes were downregulated (green dot, Figure 6A), which correlated with FDX1 expression. Of these genes, the top 50 co-expressed upregulated and downregulated genes were visualised in a heatmap (Figure 6B). Through the KEGG analysis of enriched genes, signalling pathways were ordered by normalised enrichment scores (Figure 6C). The pathway most enriched upregulated was “oxidative phosphorylation” (blue bar), and the most strongly downregulated was the “Notch signalling pathway” (orange bar) (Figure 6C).

The functional enrichment networks involved in the categories of BP, CC, and MF were plotted using LinkOmics (Supplementary Figure S2). BP analysis FDX1 co-expressed genes were mainly involved in mitochondrial gene expression, mitochondrial respiratory chain complex assembly, and various other metabolic processes, including nucleoside phosphate, tricarboxylic acid, and fatty acid (Supplementary Figure S2A). CC analysis confirmed that FDX1 co-expressed genes were enriched in the mitochondrial inner membrane, whereas MF analysis annotated the function of electron transfer activity (Supplementary Figures S2B,C).

For a more comprehensive description of BP, the event hierarchy pathways in Reactome for the top 100 upregulated co-expressed genes with FDX1 are presented as fireworks-style diagrams in Figure 6D. Reactome analysis of FDX1 co-upregulated genes demonstrated that they were mainly enriched in pathways involved in metabolism, cellular response to stimuli, organelle biosynthesis, and maintenance (Figure 6D). These pathways include “Metabolism,” “Respiratory electron transport, ATP synthesis by chemiosmotic coupling, and heat production by uncoupling proteins,” “The citric acid (TCA) cycle and respiratory electron transport,” “Complex I biogenesis” “Insulin receptor recycling,” and “Amino acids regulate mTORC1” (Details in Supplementary Table S1).

### Protein-protein interaction network of FDX1

**FIGURE 7:**
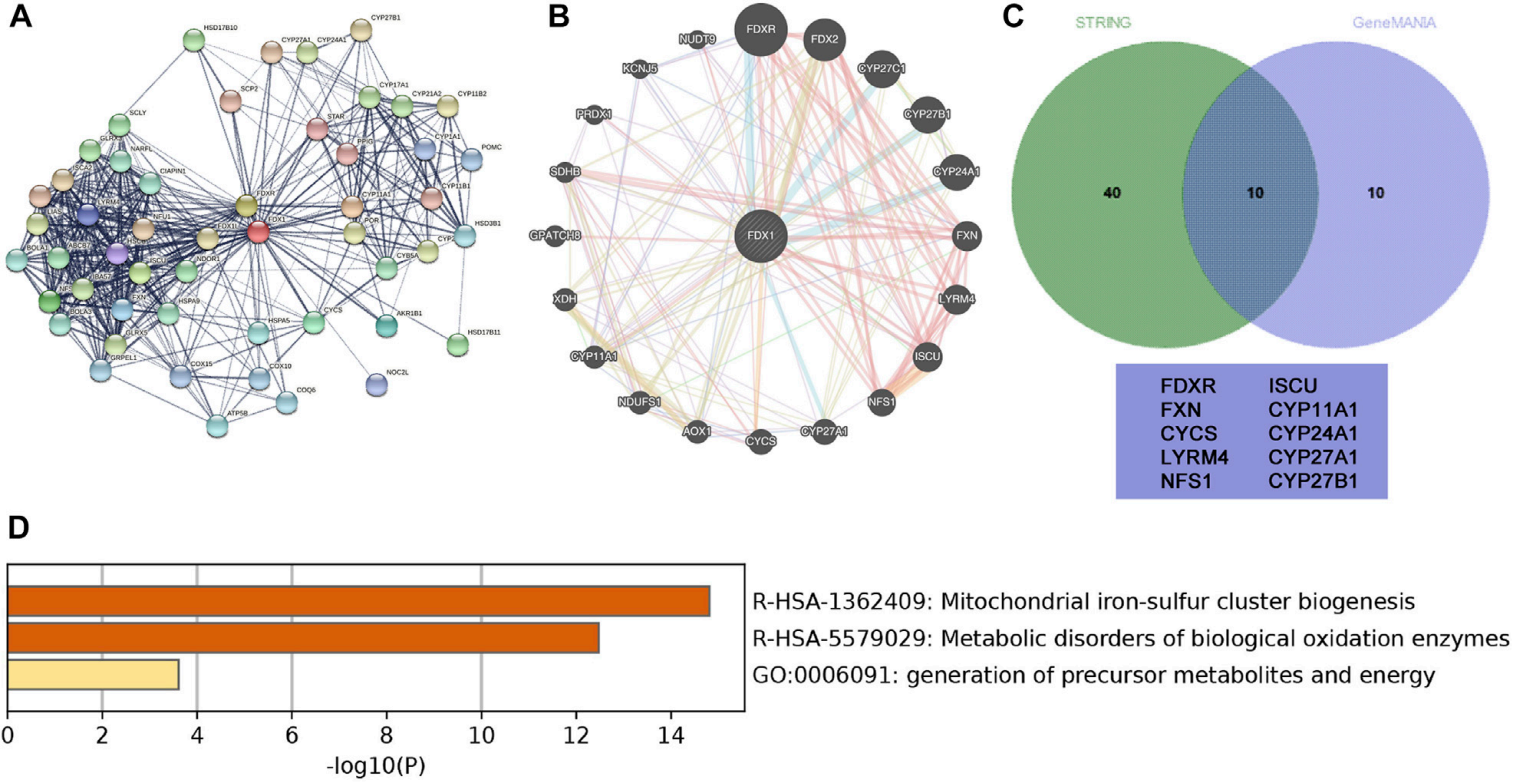
Protein-protein interaction (PPI) network of FDX1 and related genes. **(A)** STRING-created PPI network analysis of the FDX1 gene **(B)** GeneMANIAC-created PPI network analysis of the FDX1 gene. **(C)** The Interactive Venn diagram depicts the overlapping interactive 10 proteins with FDX1 based on GeneMANIA and STRING. **(D)** Functional analysis of the interactive 10 proteins using a bar diagram.

Protein-PPI networks comprise proteins that interact and play a fundamental role in signal transduction, gene expression regulation, energy metabolism activity, and cellular organisation maintenance (Braun and Gingras, 2012). To obtain proteins interacting with FDX1, we used STRING combined with GeneMANIA datasets to depict the PPI network. STRING displayed 50 proteins to exhibit a network around FDX1 with an average local clustering coefficient of 0.775 and an enrichment *p*-value of <1.0E−16 (Figure 7A). GeneMANIA displayed 20 proteins predicted to be functionally related to FDX1 (Figure 7B). Furthermore, when combining the two datasets, GeneMANIA and STRING, the 10 overlapping proteins in the Venn diagram (Figure 7C) that interacted with FDX1 were FDXR, CYCS, LYRM4, NFS1, ISCU, and cytochrome P450 family proteins (CYP11A1, CYP27A1, CYP24A1, and CYP27B1). FDX1 interactive proteins were consistent with reports that FDX1 can transfer electrons from adrenodoxin reductase to CYP11A1 and catalyse cholesterol side-chain cleavage (Sheftel et al., 2010; Strushkevich et al., 2011). Furthermore, KEGG and GO analyses demonstrated enriched functions of the 10 interactive proteins in mitochondrial iron-sulphur cluster biogenesis, metabolic disorders of oxidation enzymes, and the generation of precursor metabolites and energy in ccRCC (Figure 7D). The PPI analysis indicated that downregulated FDX1 expression in ccRCC may contribute to mitochondrial biogenesis disorders, metabolic disorders, and oxidative stress.

### Association between FDX1 and the abundance of tumour-infiltrating lymphocytes (TILs)

**FIGURE 8:**
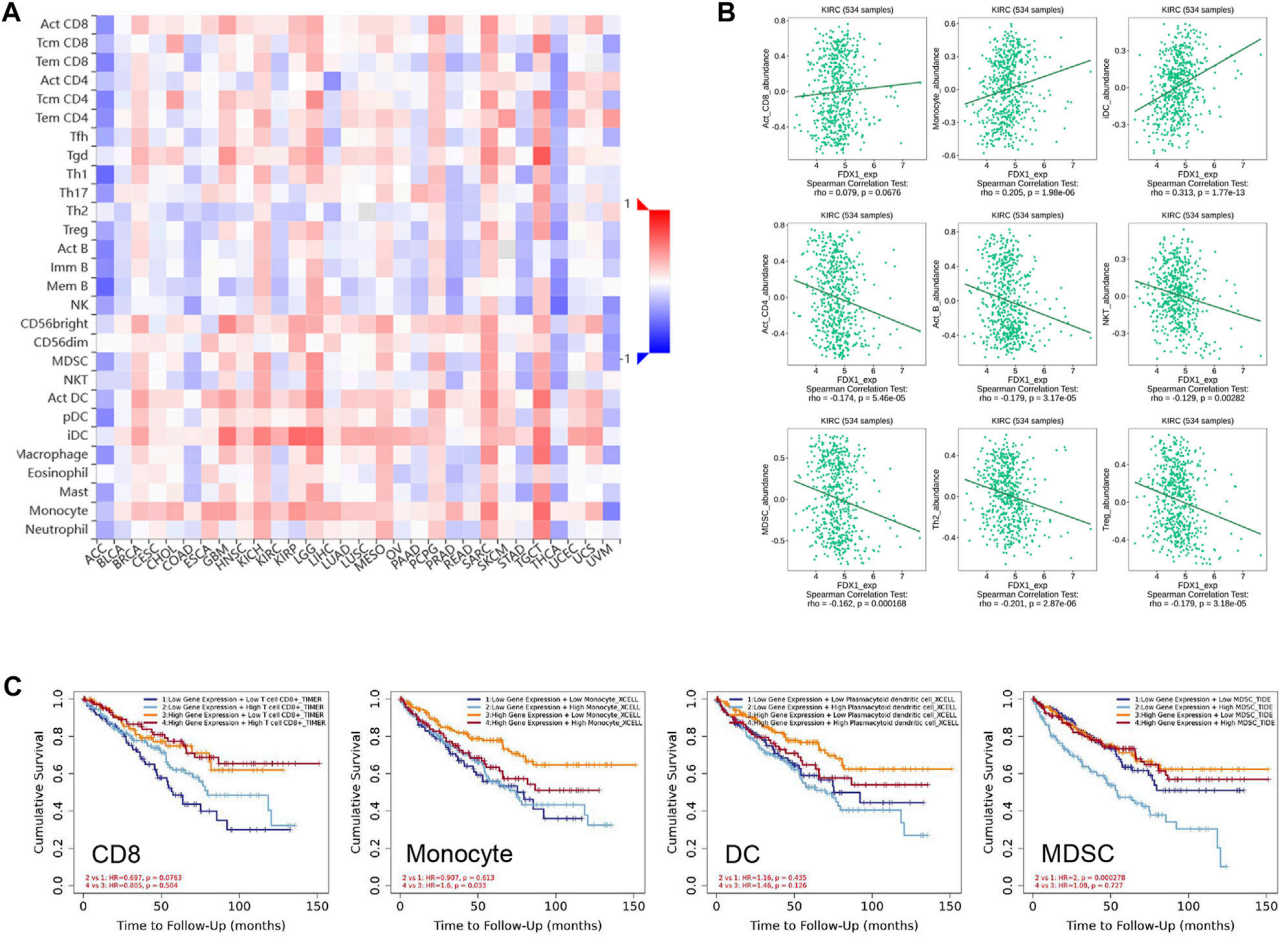
Relationship between FDX1 expression and the abundance of tumour-infiltrating lymphocytes. **(A)** Heatmaps demonstrating the positive (red colour) or negative (blue colour) correlation between FDX1 and the infiltration level of indicated immune cells across human cancers. The shade of colour in the heatmap represents the size of rho values. **(B)** Correlation of FDX1 expression and tumour-infiltrating lymphocytes like activated CD4, activated CD8, activated B, monocytes, dendritic cells, natural killer T, type 1 T helper, type 2 T helper, and regulatory T cells in 534 KIRC samples based on the Spearman correlation test. **(C)** Survival analysis grouped by different FDX1 mRNA expression level and infiltrating levels of CD8, monocyte, DC, and MDSC (Myeloid derived suppressor cell) in KIRC.

Emerging evidence suggests that the tumour microenvironment pattern and clinicopathological features of ccRCC are correlated with the prognosis and response to anti-angiogenic agents targeting VEGFR and immune checkpoint inhibitors (Kim et al., 2021). To understand the role of FDX1 in modulation of the immune microenvironment in ccRCC, we analysed the Spearman correlations between FDX1 expression and TILs using the TISIB database. The heatmap profiled the correlations between FDX1 expression and the infiltration of 28 immune cell types across human cancers (Figure 8A). In particular, FDX1 expression and TILs were negatively correlated in ACC and THCA, but positively correlated in LGG, TGCT, and SARC. There was a positive correlation between FDX1 expression and the abundance of infiltrating lymphocytes in activated CD8 + cells, monocytes, and immature dendritic cells (Figure 8B). However, FDX1 expression was negatively correlated with the infiltration of immune cells, including activated CD4, B, natural killer T, myeloid-derived suppressor cells, regulatory T cells, and type 2 T helper cells (Figure 8C). Furthermore, we investigated the impact of TILs and FDX1 expression levels on prognosis (Figure 8C). Here, the Kaplan–Meier curve revealed that the prognosis of patients with ccRCC with high expression of FDX1 improved with high levels of CD8 infiltration, low DC, low monocyte, or low levels of Myeloid-derived suppressor cell (MDSC) infiltration (Figure 8C). These results illustrate that FDX1 expression was correlated with the tumour-immune microenvironment, and the combination of FDX1-correlated tumour-infiltrating immune cells helped stratify risk and predict prognosis in ccRCC.

## Discussion

Human mitochondria contain abundant FDX1, which exerts classical functions in steroidogenesis, Fe-S cluster biosynthesis, and mitochondrial CYP enzyme reduction (Sheftel et al., 2010; Cai et al., 2017). However, the evidence supporting the role of FDX1 in carcinogenesis is limited. Furthermore, the expression profile, prognostic value, and biological function of FDX1 in ccRCC remain to be investigated.

Through bioinformatic data mining in 33 types of TCGA cancer, we found that FDX1 mRNA expression was significantly downregulated in ccRCC. We validated this finding by performing a qRT-PCR analysis of 27 pairs of tissues from patients and demonstrated a consistent decrease in FDX1 mRNA expression in tumour tissues. Furthermore, proteomic analysis of 11 TCGA cancers highlighted distinctive FDX1 expression in ccRCC, revealing the underlying substantial function involved in tumourigenesis. We then verified FDX1 protein expression by immunoblotting assay with nine pairs of tumours and adjacent tissues and by IHC in tissue microarrays with 199 pairs of tissues. Immunoblotting revealed pervasive downregulation of FDX1 expression in primary tumour lesions. Similarly to the immunoblot results, immunohistochemistry of tissue microarrays indicated significant decreases in the intensity and percentage of staining in the tumour lesion. In normal kidney tissue, intense FDX1 staining was pervasive in the renal tubule, especially in the proximal convoluted tubule (PCT). Proximal tubular cells composed of 13 distinctive clusters of epithelial cells are believed to be the most likely pathogenic origin in numerous cases of ccRCC tumourigenesis (Frew and Moch, 2015; Young et al., 2018). Intense oxygen, ions, and nutrient exchange gradients around the proximal tubule predispose individuals to developing oxidative stress and genome instability (Jonasch et al., 2021). Therefore, the characteristic location of FDX1 in PCT and the reduction of aberrant expression in ccRCC support our hypothesis that FDX1 may be involved in the carcinogenesis of ccRCC.

Besides its specific expression and localisation, FDX1 can be a novel prognostic biomarker for ccRCC. By following up our cohort of 199 cases used for TMA, prolonged 5-year OS and DFS outcomes were achieved in the high FDX1 expression group, with significant differences between the groups by Kaplan–Meier survival analysis. Multivariate Cox regression analysis identified high FDX1 expression as an independent prognostic factor for OS and DFS. When associated with clinicopathological characteristics, other common hazard factors were observed, including distal tumour metastasis, AJCC stage, and ISUP grade of ccRCC. These findings indicate FDX1 may participate in tumour progression, which is consistent with the results of the TCGA analysis.

PPI networks with the identification of 10 interactive FDX1 proteins and KEGG analysis provided information on the potential biological function of FDX1. Several BP related to FDX1 are involved in the electron transport chain, iron-sulphur protein biosynthesis, mitochondrial apoptosis, mitochondrial, and cytosolic iron homeostasis, and oxidative phosphorylation. Furthermore, the role of FDX1 in ccRCC immune infiltration was investigated and FDX1 expression levels were associated with the infiltration of multiple immune cells. Further experiments are necessary to validate its function in cancer development.

Studies conducted with siRNA knockdown of FDX1 in lung adenocarcinoma reported that neither tumour proliferation inhibition nor apoptosis or cell cycle arrest were induced (Zhang et al., 2021). However, FDX1 deficiency results in multiple metabolic alterations, including fructose 6-phosphate accumulation, promotion of fatty acid oxidation, inhibition of L-cysteine and increased abundance of L-glutamine (Zhang et al., 2021). Therefore, FDX1 may be involved in metabolic dysregulation in carcinogenesis, which could change the phenotype and tumour microenvironment. This metabolic reprogramming tested in LUAD provides clues to the function of the FDX1 gene, especially in RCC. The molecular features of RCC are modulated by different genes, including VHL, MET, FLCN, TSC1, TSC2, FH, TFE3, TFEB, MITF, SDH, and PTEN, to alert various aspects of metabolism (Linehan et al., 2010; Linehan and Ricketts, 2013). Clear cell renal cell carcinoma, the most common form of RCC, is characterised by fatty acid and glycogen reprogramming that rewires this aberrant metabolism in ubiquitous ontology events of chromosome 3p deletion and VHL mutation or deletion (Du et al., 2017; Wettersten et al., 2017; Jonasch et al., 2021). Inactivation of VHL inactivation stabilises HIF1α and HIF2α transcription factors, which activate downstream pathways to regulate iron metabolism [36], angiogenesis (vascular endothelial growth factor and platelet-derived growth factor), glycolysis (pyruvate dehydrogenase kinase 1 [37]), lipid disposition (carnitine palmitoyl transferase 1A [5]), and other processes that drive cancer progression. By interacting with mitochondrial function, metabolic reprogramming in ccRCC cells can identify new biomarkers and develop new ion-target therapeutic approaches.

FDX1 has recently been reported to be a direct target of elesclomol copper ionophores. The presence of copper determines the response to copper-RCD in cancer treatment (Tsvetkov et al., 2022). Copper has emerged as a significant mineral nutrient and plays a fundamental role in metal signalling modulation, metalloallosteric regulation, mitochondrial respiration, antioxidant defence, and neurotransmitters and determines cell fate by reprogramming metabolism (Ruiz et al., 2021; Ge et al., 2022). Cellular copper overload can cause enhanced proliferation and growth defined as “Cuproplasia.” Compared to healthy tissues, cancer progression increases the dependency on cellular Cu concentration for proliferation, angiogenesis, and metastasis (Blockhuys et al., 2017). Higher copper demand has been observed in the serum and solid tumours of patients with different tumour types, including renal cell carcinoma (Abdel-Gawad et al., 2020), oesophageal and gastric cancer (Yaman et al., 2007; Sohrabi et al., 2021), colorectal cancer (Sohrabi et al., 2018), and lung cancer (Bai et al., 2019).

Therefore, the high abundance of Cu in tumour cells can be exploited in the development of anti-cancer therapy by administering Cu chelators to suppress cuproplasia in tumour proliferation, progression, and metastasis by modulating mitochondrial metabolism (Cui et al., 2021; Ramchandani et al., 2021). Contrary to the depletion of the copper concentration in cells, aberrant accumulation of cellular copper by copper ionophores is also an effective alternative to induce cellular toxicity in RCD, termed cuproptosis (Tsvetkov et al., 2022). Various classes of copper ionophores apply to cancer, including disulfuram, elesclomol, bis(thiosemicarbazone) analogues, 8-hydroxyquinolines, and flavones (Oliveri, 2022). The intervention with disulfiram and temozolomide in patients with glioblastoma after standard chemoradiotherapy possesses an acceptable safety profile and has had promising PFS in a phase I study (Huang et al., 2016; Huang et al., 2018). Various types of copper chelators have been selected as anti-cancer agents, such as D-penicillamine, trientine, ATN-224, and tetrathiomolybdate (TTM). In advanced kidney cancer with TTM treatment in a phase II trial, stable disease was achieved in 31% of patients, and proangiogenic molecules of interleukin-6, interleukin-8, vascular endothelial growth factor, and basic fibroblast growth factor in serum decreased to realise anti-angiogenic effects (Redman et al., 2003). Furthermore, the development of copper-depleting nanoparticles combined with copper-depleting moieties and semi-conducting polymer nanoparticles to treat triple-negative breast cancer shows high efficacy and safety in mice and offers clinical potential for cancer intervention (Cui et al., 2021).

This study has limitations that can be overcome by further research. First, the public bioinformatics database was limited to comprehensive clinical and pathological information. In particular, the underlying mechanism of FDX1 in ccRCC remains to be explored *in vivo* or *in vitro* using FDX1 overexpression/knockdown clone construction experiments. Second, validation of the enriched biological function and modelling of the related pathway by functional enrichment analysis of FDX1 was not performed, but is necessary, especially that of the metabolic pathway regulated by mitochondria. We did not acquire complete information on patients’ lactate dehydrogenase (LDH) levels. Low plasma levels of LDH reflect a higher biological dependency on mitochondrial metabolism than glycolysis. Therefore, these patients display increased sensitivity to copper ionophores for elevated lipoylated proteins regulated by FDX1 expression (Tsvetkov et al., 2022). It is unknown whether there is a correlation between LDH levels and FDX1 abundance in relation to clinicopathological malignancy. Last, there is an urgent need to determine another effective cuproptosis-related signature as both a prognostic and diagnostic biomarker for ccRCC, and as a potential target for small molecule compound development.

In this study, we explored the expression and prognostic value of FDX1 in numerous types of cancer and identified its characteristic role in ccRCC using a series of online bioinformatic databases. The experimental validation based on the database-generated inference was validated *in vitro* using clinical samples from tissue microarrays and cell lines. FDX1 was significantly downregulated in ccRCC at the mRNA and protein level and could be a potential prognostic biomarker for patients with ccRCC. Functional enrichment analysis and the PPI network revealed the crucial function of FDX1, which is involved in mitochondrial metabolism disorders besides iron-sulphur cluster biogenesis in ccRCC. FDX1 may influence TILs, and promoter hypomethylation status indicates a worse prognosis. More multifaceted *in vitro* and *in vivo* experimental studies are necessary to validate the role of FDX1 MF intervention in metabolic reprogramming and tumourigenesis of ccRCC.

## Conclusion

FDX1 mRNA and protein expression were aberrantly downregulated and associated with malignant progression and advanced clinicopathological characteristics of ccRCC, whereas in adjacent non-tumour kidney tissue, it was abundantly expressed and cytoplasmically localised in the PCT. FDX could serve as a promising prognostic biomarker to stratify patients with ccRCC. Based on functional annotations, further mechanistical investigation of these findings could deepen our understanding of the role of FDX1 in ccRCC tumorigenesis.

## Data Availability

The original contributions presented in the study are included in the article/Supplementary Material, further inquiries can be directed to the corresponding authors.

## Supplementary material

Supplementary Figure S1 FDX1 mRNA and protein expression across cancer cell lines.

Supplementary Figure S2 The functional enrichment networks of FDX1 co-expressed genes involved in the BP, CC, and MF.

Supplementary Table S1 Reactome pathway analysis of FDX1 co-upregulated genes.
