# Supplementary figures and images for: FDX1 expression predicts favourable prognosis in clear cell renal cell carcinoma identified by bioinformatics and tissue microarray analysis
Source: Front Genet. 2022 Sep 16;13:994741. doi: 10.3389/fgene.2022.994741 (PMC9523472; doi:10.3389/fgene.2022.994741)

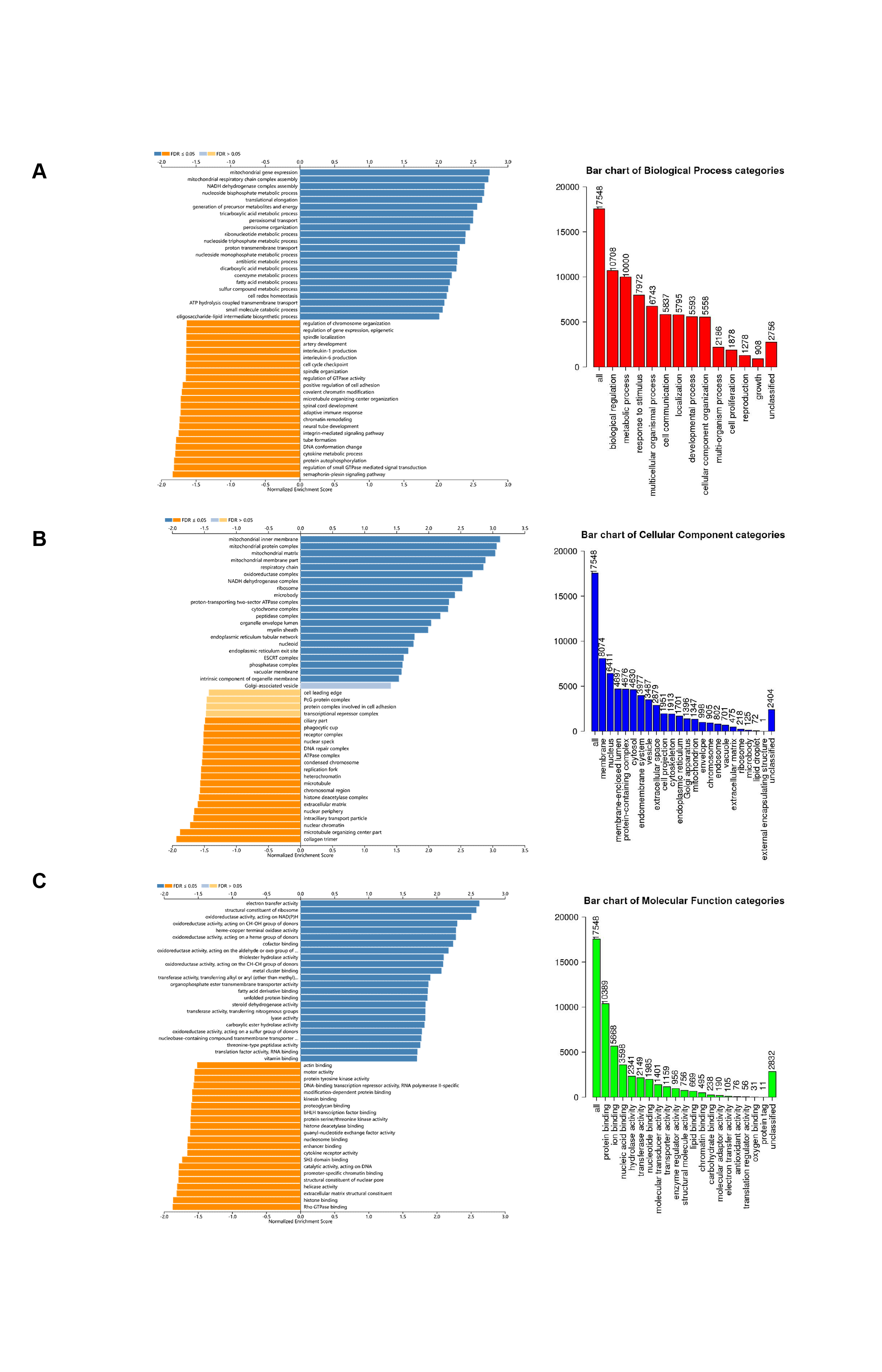

Supplement: Supplementary file 1 [file Image2.TIF]

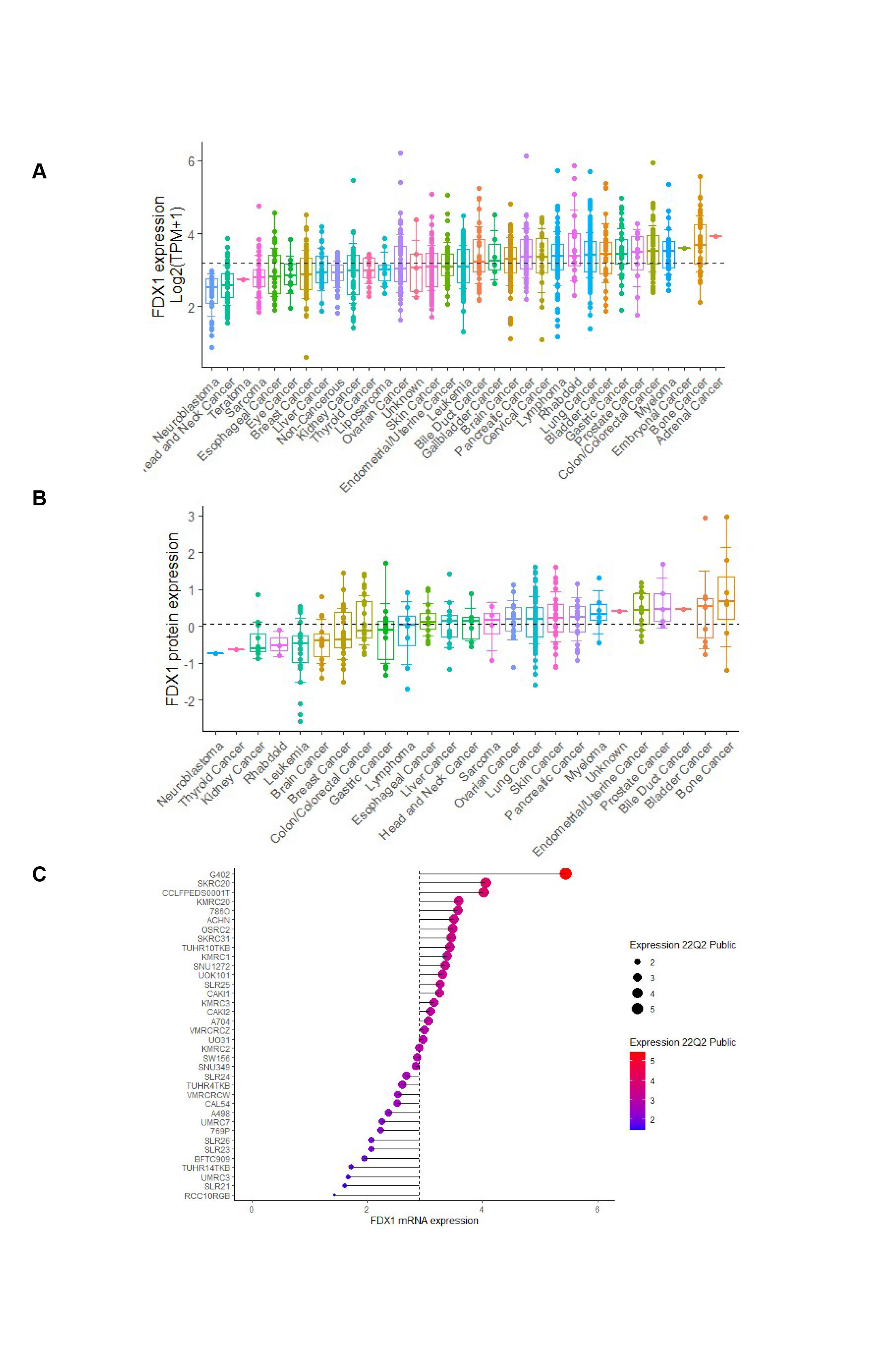

Supplement: Supplementary file 2 [file Image1.TIF]
